# Impact of prognostic nutritional index and geriatric nutritional risk index on prognosis in elderly patients with early-stage prostate cancer

**DOI:** 10.3389/fnut.2026.1745718

**Published:** 2026-02-17

**Authors:** Pinar Peker, Asli Geçgel, Oǧuzcan Özkan, Serdar Yilmaz, Seher Selvi, Cafer Cirik, Sezen Koca, Alpay Düşgün, Burcu Arslan Benli, Berna Bozkurt Duman, Timuçin Çil

**Affiliations:** 1 Department of Medical Oncology, Adana City Training and Research Hospital, Adana, Türkiye; 2 Department of Medical Oncology, Yozgat City Hospital, Yozgat, Türkiye; 3 Department of Medical Oncology, Usak Training and Research Hospital, Usak, Türkiye

**Keywords:** early-stage prostate cancer, elderly patients, geriatric nutritional risk index, prognostic nutrition index, survival analysis

## Abstract

**Aim:**

Prostate cancer predominantly affects older men and generally has a favorable early-stage prognosis, yet the prognostic significance of nutritional and inflammatory status remains uncertain. This study evaluated the prognostic value of the Prognostic Nutritional Index (PNI) and Geriatric Nutritional Risk Index (GNRI) in elderly patients with localized prostate cancer.

**Methods:**

This single-center retrospective cohort study included 205 patients aged ≥65 years with early-stage prostate cancer treated between 2018 and 2024. Nutritional status was assessed at baseline using serum albumin, lymphocyte count, and body weight to calculate the PNI and GNRI. Overall survival was analyzed using standard survival analysis methods. All statistical analyses were performed using SPSS software version 26.0.

**Results:**

The median patient age was 72 years. Of all patients, 41% were 75 years or older. Survival analysis showed that patients with low PNI had a median OS of 78 months. Those with high PNI had a median OS of 115 months (*p* = 0.008). Low GNRI was linked to a median survival of 74 months. High GNRI was linked to 120 months (*p* = 0.009). Higher Gleason score (≥8), higher PSA (≥10 ng/mL), older age (≥75 years), and clinical T2 disease were associated with worse outcomes. By contrast, radiotherapy improved survival (122 vs. 94 months, *p* = 0.008). In multivariate analysis, low PNI, low GNRI, high Gleason score, and high PSA remained independent predictors.

**Conclusions:**

The PNI and GNRI serve as practical and accessible indicators of nutritional status and inflammation, thereby improving prognostic assessment and risk stratification in older patients with early-stage prostate cancer.

## 1 Introduction

Prostate cancer (PCa) is a common cancer in men and a major global health burden. It ranks as the second most prevalent cancer worldwide (1). Early-stage PCa, when the tumor is confined to the prostate, usually has a favorable prognosis (2). Routine prostate-specific antigen (PSA) screening and longer life expectancy have increased the number of early-stage diagnoses (3).

However, outcomes among patients with early-stage PCa vary considerably. This heterogeneity reflects differences not only in tumor biology, but also in host-related factors such as comorbidities, nutritional status, and systemic inflammation (4). In elderly patients in particular, age-associated immune dysregulation and malnutrition may accelerate cancer progression and influence treatment tolerance and survival. Malnutrition in elderly cancer patients can activate systemic inflammation, which may contribute to tumor progression and negatively influence treatment tolerance and outcomes (5). Therefore, in older individuals with prostate cancer, management should not rely solely on tumor stage but also incorporate the patient's overall health and nutritional status. In some early-stage cases—particularly among frail patients or those with significant comorbidities—active surveillance may be appropriate instead of immediate treatment (6). The Prognostic Nutritional Index (PNI) and the Geriatric Nutritional Risk Index (GNRI) are simple biochemical indices that reflect both nutritional reserve and immune competence (7). These markers, derived from serum albumin, lymphocyte count, and body weight parameters, have been increasingly recognized as predictors of clinical outcomes in several malignancies (8–11). Importantly, lower PNI or GNRI levels indicate impaired nutritional and immune function and have consistently been associated with worse survival in elderly cancer populations (12, 13). Frailty, which is common in older cancer patients, further increases vulnerability to treatment-related toxicity and mortality. This underscores the importance of including these indices in clinical assessment.

Several studies have highlighted the potential of the GNRI as a frailty indicator, especially in older surgical patients. Lower GNRI scores have been linked to increased postoperative complications, prolonged hospitalization, and higher mortality risk (14). The GNRI integrates nutritional and inflammatory status, offering a practical way to identify physiologic vulnerability in elderly cancer patients (15).

Despite growing evidence for their prognostic value in other malignancies, the role of PNI and GNRI in early-stage prostate cancer remains insufficiently explored. Incorporating these indices into clinical assessment may help refine prognostic stratification by capturing host-related vulnerabilities not reflected in tumor-based parameters alone.

Therefore, we assessed whether the GNRI and the PNI were predictive of a better early-stage prognosis in older patients with PCa. To the best of our knowledge, no comparison study comparing the prognostic surveillance power of PNI and GNRI in a cohort of elderly patients with early-stage prostate cancer has been published to date. Our analysis examines whether these host-related parameters provide prognostic information beyond well-known tumor-associated factors such as PSA, Gleason score, and clinical tumor stage, in contrast to earlier studies that were primarily conducted in the metastatic setting or assessed individual nutritional markers.

## 2 Material and method

### 2.1 Study design and ethical approval

This study was a retrospective, observational cohort study conducted at Adana City Hospital. Investigators from Yozgat City Hospital and Usak Training and Research Hospital joined in statistical analyses and independently validated the results. However, no patient data were collected from these centers. All clinical, laboratory, and survival data came exclusively from Adana City Training and Research Hospital. Thus, despite external collaboration, the study is a single-center retrospective cohort. Ethical approval was obtained from the Adana City Hospital Ethics Committee (Approval date: 10/04/2025; Meeting number: 12; Decision no: 475). The study was carried out in accordance with the principles of the Declaration of Helsinki.

### 2.2 Patient selection

A total of 205 male patients aged 65 years or older, diagnosed with early-stage prostate cancer (clinical stage T1–T2 without regional or distant metastasis) between January 2018 and December 2024, were included in the study. Patients were excluded if they had metastatic disease, previous or synchronous malignancies, or incomplete baseline clinical or laboratory data.

### 2.3 Data collection and variables

Baseline demographic, clinical, and pathological data including age, PSA level, Gleason score, tumor stage, histological subtype, comorbidities, and nutritional indices (PNI, GNRI) were collected from electronic medical records.

### 2.4 Nutritional indices

Two nutrition-related prognostic indices were calculated:

PNI = 10 × serum albumin (g/dL) + 0.005 × total lymphocyte count (/μL)

GNRI= 14.89 × albumin (g/dL) + 41.7 × (actual weight/ideal body weight) Ideal body weight was estimated using the Lorentz formula (16).

### 2.5 Statistical analysis

Statistical analyses were conducted using SPSS version 26.0 (IBM Corp., Armonk, NY, USA). For continuous variables, normality was assessed with the Kolmogorov–Smirnov test, and results are presented as mean ± standard deviation or median (interquartile range), as appropriate. Categorical variables are presented as counts and percentages.

Overall survival (OS) was defined as the time from diagnosis to death or last follow-up. Median follow-up duration was estimated using the reverse Kaplan–Meier method. Survival curves were generated using the Kaplan–Meier method and compared with the log-rank test.

Prespecified subgroup analyses were conducted based on age, PSA level, Gleason score, clinical tumor stage, and radiotherapy status.

Univariate and multivariate Cox proportional hazards regression analyses were performed to identify factors associated with OS. Variables with p <0.05 in univariate analyses and those deemed clinically relevant were included in the multivariate model using a forced-entry method. Results are presented as hazard ratios (HRs) with 95% confidence intervals (CIs). The proportional hazards assumption was evaluated using Schoenfeld residuals, and multicollinearity was assessed using variance inflation factors (VIFs).

Optimal cut-off values for nutritional indices were determined using receiver operating characteristic (ROC) curve analysis with the Youden index. Correlations between nutritional indices and tumor-related parameters were evaluated using Spearman's rank correlation coefficients. A two-sided *p*-value <0.05 was considered statistically significant.

## 3 Results

A total of 205 elderly patients with early-stage prostate cancer were included in the analysis. The median age at diagnosis was 72 years (IQR: 68–78), and 41% of the cohort (*n* = 84) were aged ≥75 years. Regarding nutritional status, the mean PNI and GNRI values were 47.5 ± 6.1 and 96.4 ± 8.7, respectively. The median baseline PSA level was 9.8 ng/mL (range: 4.2–28.5 ng/mL). Most patients had a Gleason score <8 (64.4%) and T1 stage disease (57.6%).

In terms of histology, acinar adenocarcinoma was the predominant subtype (95.1%). Perineural invasion and lymphovascular invasion were identified in 22.9 and 15.6% of cases, respectively. The most common comorbidities were hypertension (44.9%) and diabetes mellitus (32.2%). The mean BMI was 26.1 ± 3.4 kg/m^2^. ECOG performance status was 0–1 in most patients (88.8%). Baseline demographic, clinical, pathological, and nutritional characteristics of the cohort are summarized in Table 1.

**Table 1:** Baseline demographic, clinical, pathological, and lifestyle characteristics of elderly patients with early-stage prostate cancer included in the study cohort (*n* = 205).

**Variable**	**Value**
Total number of patients	205
Age (years)	72 (IQR: 68–78)
PNI	47.5 ± 6.1
GNRI	96.4 ± 8.7
PSA (ng/mL)	9.8 (4.2–28.5)
Gleason Score <8/≥8	132 (64.4%)/73 (35.6%)
Tumor Stage T1/T2	118 (57.6%)/87 (42.4%)
Radiotherapy (Yes/No)	116 (56.6%)/89 (43.4%)
Histological subtype	Acinar adenocarcinoma: 195 (95.1%)/Others: 10 (4.9%)
Perineural invasion	Present: 47 (22.9%)/Absent: 158 (77.1%)
Lymphovascular invasion	Present: 32 (15.6%)/Absent: 173 (84.4%)
Hypertension	92 (44.9%)
Diabetes mellitus	66 (32.2%)
Cardiovascular disease	48 (23.4%)
BMI (kg/m^2^)	26.1 ± 3.4
ECOG Performance Status 0–1/≥2	182 (88.8%)/23 (11.2%)
Family history of PCa	38 (18.5%)
Smoking (Current/Former/Never)	69 (33.7%)/136 (66.3%)
Alcohol use (Regular/Never)	41 (20.0%)/164 (80.0%)
Prior surgery	74 (36.1%)
Androgen deprivation therapy (ADT)	83 (40.5%)

Continuous variables are presented as mean ± SD or median (IQR), and categorical variables are expressed as frequencies and percentages.

The median follow-up duration, calculated using the reverse Kaplan–Meier method, was 41.2 months (95% CI: 37.6–44.8). Notably, patients with low PNI (<45) had significantly shorter median overall survival than those with higher PNI (78 vs. 115 months; *p* = 0.008), and a similar association was observed for GNRI (<92 vs. ≥92: 74 vs. 120 months; *p* = 0.009).

Additionally, worse survival outcomes correlated with clinical stage T2, Gleason score ≥8, PSA ≥10 ng/mL, age ≥75 years, and the absence of radiotherapy. Among patients who underwent radiotherapy, median survival was longer than among those who did not (122 vs. 94 months; *p* = 0.008). In the multivariate Cox regression analysis, low PNI, low GNRI, high Gleason score, elevated PSA, T2 stage, age ≥75, and lack of radiotherapy all remained independent predictors of mortality (all *p* < 0.05). The proportional hazards assumption was assessed using Schoenfeld residuals, and no significant violations were observed for any covariate or for the global model (all *p* > 0.10); thus, the Cox regression assumptions were satisfied.

The median PNI and GNRI values were 48.2 (IQR: 44.5–52.6) and 95.4 (IQR: 89.2–101.8), respectively. In the overall cohort, 62 patients (30.2%) had low PNI (<45), and 58 patients (28.3%) had low GNRI (<92). Correlation analyses showed that neither PNI nor GNRI was significantly correlated with PSA levels or Gleason score.

**Figure 1:**
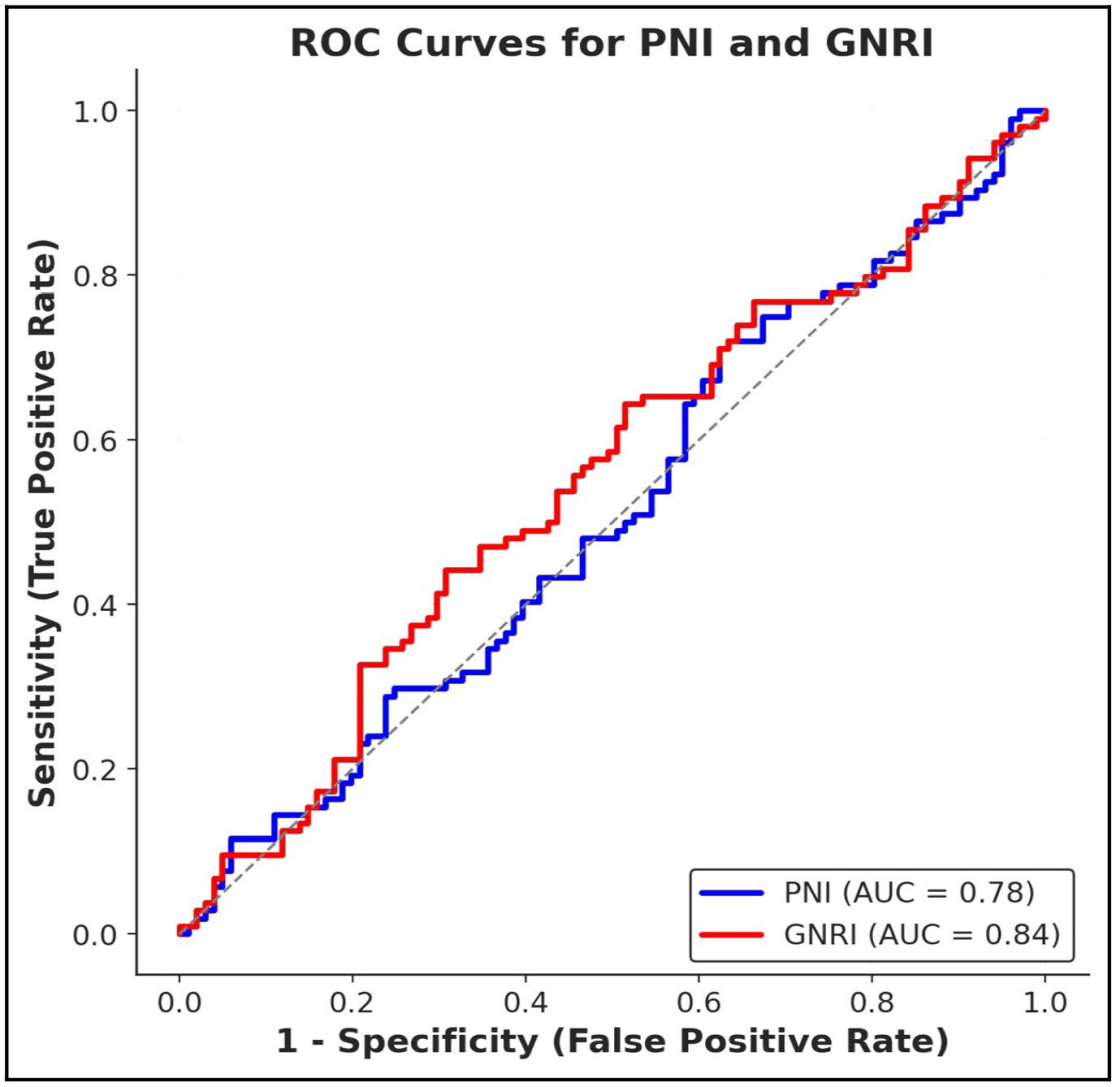
ROC curves for PNI and GNRI, demonstrating their predictive performance for overall survival. PNI: AUC = 0.78, sensitivity = 72%, specificity = 75%; GNRI: AUC = 0.84, sensitivity = 78%, specificity = 80%.

Over the follow-up period, 51 deaths occurred and were included as endpoint events in the ROC analyses. ROC curve analysis demonstrated acceptable discriminatory ability for overall survival prediction: the AUC was 0.78 for PNI (sensitivity: 72%, specificity: 75%) and 0.84 for GNRI (sensitivity: 78%, specificity: 80%), indicating slightly greater prognostic accuracy for GNRI. Log-rank tests supported significant survival differences between high and low index groups for both PNI (χ^2^ = 6.89, *p* = 0.008) and GNRI (χ^2^ = 6.72, *p* = 0.009), as shown in Figure 1.

**Figure 2:**
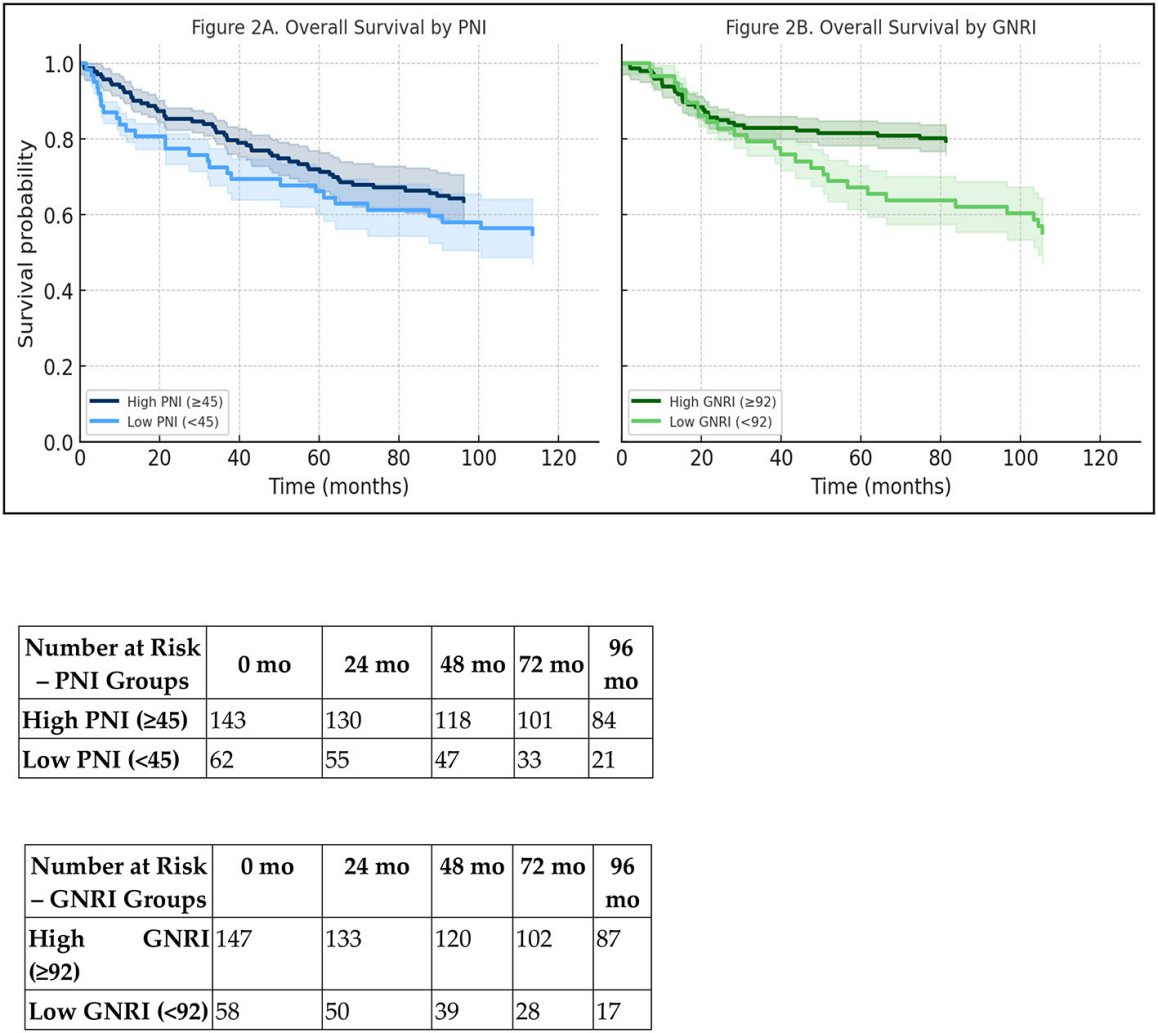
Kaplan–Meier survival curves for overall survival in elderly patients with early-stage PCa. **(A)** Survival stratified by PNI, classified as low (<45) vs. high (≥45); **(B)** Survival stratified by GNRI, classified as low (<92) vs. high (≥92); A risk table summarizing the number of patients at risk at baseline, 24, 48, 72, and 96 months is provided below each panel for both PNI and GNRI groups.

Kaplan–Meier analyses demonstrated statistically significant differences in overall survival between the high and low PNI and GNRI groups (Figure 2). Kaplan–Meier curves stratified by PNI and GNRI were presented with 95% confidence intervals, and the number of patients at risk over time was displayed for each curve.

In the multivariate Cox proportional hazards analysis (Table 2), low PNI, low GNRI, Gleason score ≥8, PSA ≥10 ng/mL, clinical stage T2, age ≥75 years, and absence of radiotherapy were statistically significantly associated with overall survival. In multivariate models including age, Gleason score, PSA level, clinical T stage, and radiotherapy, low PNI and low GNRI remained independently associated with overall survival (Table 2).

**Table 2:** Multivariate Cox proportional hazards model evaluating independent prognostic factors for overall survival in elderly patients with early-stage prostate cancer.

**Variable**	**HR**	**95% CI**	***p*-value**
Low PNI (<45)	1.65	1.12–2.45	0.012
Low GNRI (<92)	1.78	1.25–2.64	0.004
Gleason Score ≥8	1.82	1.30–2.55	0.002
PSA ≥10 ng/mL	1.74	1.21–2.51	0.006
Tumor stage T2	1.75	1.23–2.48	0.004
No Radiotherapy	1.55	1.10–2.18	0.015
Age(>75)	1.68	1.19-2.37	0.008

Variables included in the multivariate analysis were those with *p* < 0.05 in univariate analysis or deemed clinically relevant. HRs greater than 1.0 indicate an increased risk of mortality.

Subgroup analyses by age, PSA level, Gleason score, tumor stage, and radiotherapy showed distinct survival patterns. Median overall survival was longer in patients aged 65–75 than in those 75–85 (110 vs. 85 months; *p* = 0.005). Patients who received radiotherapy survived longer than those who did not (122 vs. 94 months; p = 0.008), as shown in Figure 2.

A risk table summarizing the number of patients at risk at baseline, 24, 48, 72, and 96 months is provided below each panel for both PNI and GNRI groups.

**Figure 3:**
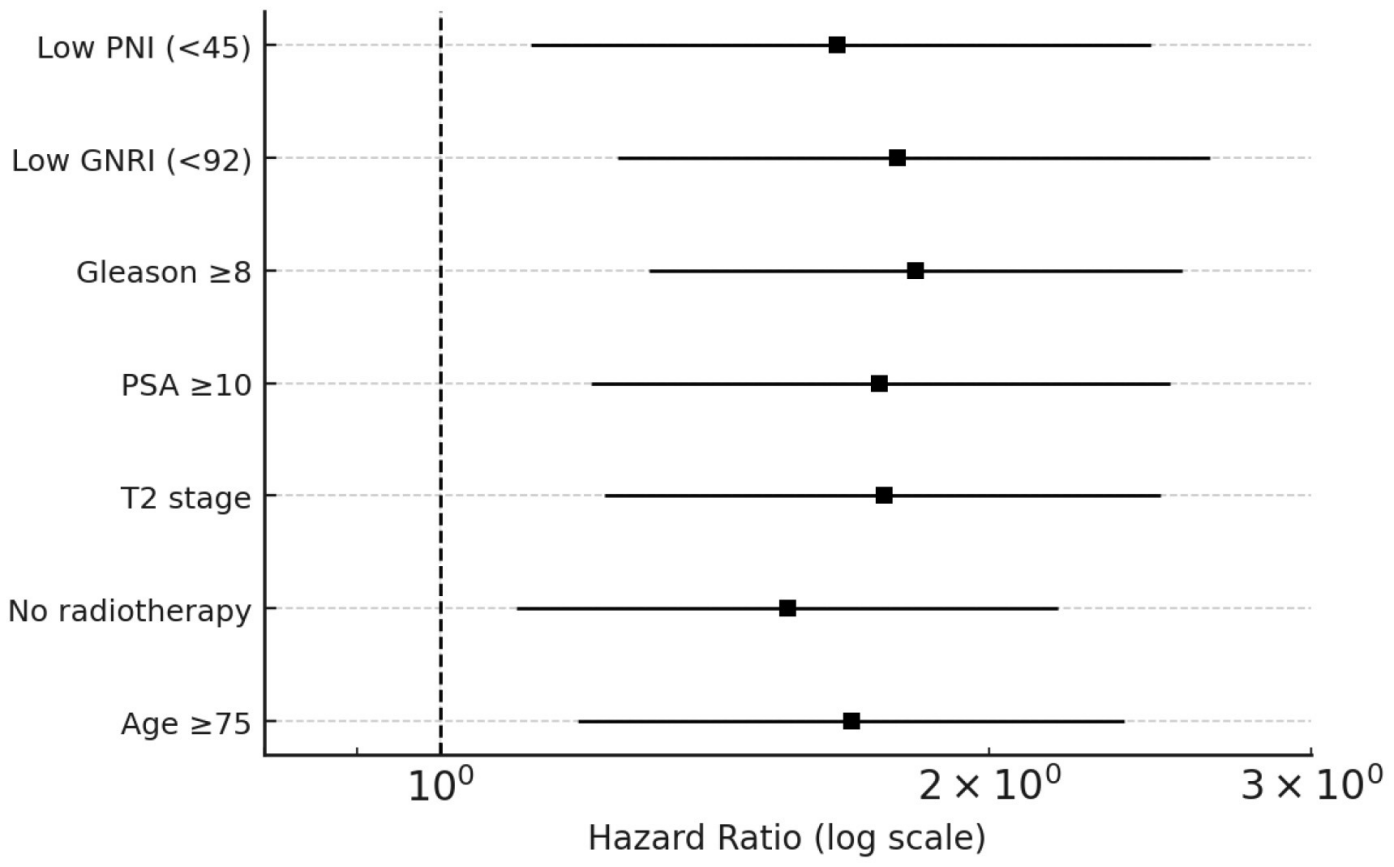
Forest plot of the multivariate Cox proportional hazards regression analysis for overall survival in elderly patients with early-stage prostate cancer. Hazard ratios (HRs) with corresponding 95% CIs are shown for each covariate included in the final model. Values to the right of the vertical reference line (HR = 1.0) indicate an increased risk of mortality, whereas values to the left indicate a protective association.

Patients with stage T2 tumors had a 1.75-fold increased risk of mortality compared with those with T1 disease (HR = 1.75, 95% CI: 1.23–2.48, *p* = 0.004), and patients who did not receive radiotherapy had a higher risk of death than those who did (HR = 1.55, *p* = 0.015). Figure 3 displays the multivariate model results as a forest plot.

## 4 Discussion

Aging is commonly associated with malnutrition and the loss of skeletal muscle mass, both of which contribute to sarcopenia, a condition linked to frailty and adverse clinical outcomes in cancer patients (17, 18). Sarcopenia, together with comorbidities, systemic inflammation, and metabolic dysregulation, diminishes physical resilience, impairs tolerance to oncologic therapies, and elevates mortality risk (19, 20). While early-stage PCa is often clinically indolent, treatment decisions for elderly patients should consider not only tumor characteristics but also host-related factors.

The prognostic significance of nutritional and inflammatory status is demonstrated by markers such as albumin and lymphocyte count, which indicate protein reserves and immune competence (21). Decreases in these parameters can impair antitumor immunity and negatively influence treatment response and survival (22). Consequently, evaluating nutrition- and inflammation-based indices yields clinically relevant information regarding patient physiologic fitness, thereby supplementing traditional tumor-based prognostic factors.

Alongside PNI and GNRI, baseline BMI, prior hormonal therapy exposure, and histopathological features are associated with prognosis in elderly patients with prostate cancer. Nevertheless, accumulating evidence suggests that BMI alone does not sufficiently capture nutritional and inflammatory status in geriatric oncology (18). Although the mean BMI in this cohort was within the overweight range, prior studies indicate that higher BMI does not consistently provide a survival benefit, as increased adiposity may conceal underlying sarcopenia and protein–energy malnutrition (23). Therefore, combining BMI with nutrition- and inflammation-based indices such as GNRI and PNI enables a more precise evaluation of nutritional risk and physiologic reserve in elderly patients.

Androgen deprivation therapy (ADT) is a standard component of prostate cancer treatment; however, it is consistently associated with adverse metabolic and body composition changes, such as increased adiposity and reduced lean muscle mass, which may worsen frailty in elderly men (24). These findings highlight the necessity of nutritional monitoring and early supportive interventions to address treatment-related sarcopenia and cachexia in patients undergoing ADT.

Integrating unfavorable prognostic factors with nutrition-based indices, including PNI and GNRI, may provide additional information for prognostic stratification by reflecting both tumor-related features and host physiological reserve (25, 26). This study comprehensively evaluated the prognostic significance of the PNI and GNRI in elderly patients with early-stage PCa. The results indicate that lower PNI and GNRI values are associated with poorer clinical outcomes. These findings suggest that host-related nutritional and immune status offer valuable prognostic information that complements traditional tumor-based prognostic factors.

While the prognostic significance of the PNI and GNRI has been extensively investigated in gastrointestinal, lung, and pancreatic cancers, evidence in PCa, particularly in early-stage disease and among elderly populations, remains scarce. Most prior studies have concentrated on metastatic prostate cancer or have assessed individual nutritional markers, such as serum albumin, the albumin-to-globulin ratio (AGR), or the neutrophil-to-lymphocyte ratio (NLR), rather than composite indices. In contrast, the present cohort comprises only elderly men with low-risk prostate cancer, a group in which frailty and nutritional status may significantly influence prognosis but are underrepresented in prognostic evaluations (27, 28). This study addresses these gaps by demonstrating that both PNI and GNRI independently predict overall survival, even after adjusting for established tumor-related prognostic factors, including PSA, Gleason score, and tumor stage.

The observed associations are likely biologically plausible due to the interplay among systemic inflammation, nutritional impairment, and tumor–host interactions. Malnutrition impairs immune function and fosters a proinflammatory environment. These conditions can facilitate tumor progression and increase treatment resistance. The GNRI incorporates serum albumin, while the PNI includes lymphocyte count. Lower values of these indices may indicate compromised antitumor immunity and diminished physiological reserve. This aligns with their association with poorer outcomes in this cohort.

Previous studies of metastatic PCa have reported inconsistent associations between nutritional parameters and prognosis, likely due to heterogeneous disease biology and confounding systemic effects associated with advanced-stage disease (29). The present results extend the findings of Zheng et al., who identified an association between low PNI and adverse survival but did not assess GNRI or conduct comprehensive multivariable adjustment. Although markers such as CRP, NLR, and AGR have been associated with disease severity, these indicators require laboratory panels that may not be universally accessible. In contrast, PNI and GNRI can be calculated from routine laboratory values, making them practical tools for geriatric risk assessment in routine oncology practice.

Takele et al. (30) demonstrated that malnutrition is closely associated with systemic inflammation, as indicated by elevated cytokine levels and impaired neutrophil function. Previous studies have established that a low pretreatment PNI correlates with worse PFS and OS in patients with PCa, thereby supporting its utility as a prognostic marker. In alignment with these findings, both low PNI and low GNRI levels were significantly associated with reduced overall survival in the present cohort. Furthermore, multivariate analyses indicated that PNI and GNRI retained prognostic significance independent of established clinical factors, such as Gleason score and tumor stage, highlighting the importance of the relationship between nutritional status and the inflammatory burden in cancer prognosis (31).

Although PNI reflects aspects of both nutritional and immune status, the absence of direct inflammatory markers such as CRP and NLR, which were not routinely measured or consistently available in this retrospective dataset, limited a comprehensive assessment of systemic inflammation (32). Consistent with previous evidence, higher PSA levels and elevated Gleason scores were associated with poorer clinical outcomes, whereas patients with T1-stage tumors and lower PSA levels demonstrated the most favorable survival profiles (33–35). Notably, elevated PSA levels predicted worse survival, particularly when combined with stage T2 disease and low PNI or GNRI values, suggesting that integrating tumor- and host-related factors may improve prognostic stratification.

Kaya et al. (36) reported that serum albumin, a component of GNRI, may provide additional prognostic insight. These findings indicate that nutritional biomarkers can complement tumor-based parameters in risk stratification. The present results support this integrated approach. Assessing tumor stage and PSA, along with PNI and GNRI, may better identify patients at increased risk of adverse outcomes.

Comparisons of subgroups defined by age, PSA level, Gleason score, and tumor stage reveal distinct prognostic gradients, highlighting the heterogeneity present in early-stage PCa among elderly patients. For instance, an older individual with a seemingly favorable tumor profile may still be classified as high-risk if a low GNRI is present, indicating reduced physiological reserve. This comprehensive assessment is especially significant when evaluating the suitability of curative treatment compared to active surveillance in elderly men (37). Additionally, recent evidence indicates that lifestyle and dietary patterns can affect nutritional status and prostate cancer risk, emphasizing the need to integrate host-related factors into clinical decision-making (38).

BMI alone has limited prognostic value in several malignancies, including renal cell carcinoma, non–small cell lung cancer, small cell lung cancer, and gastric cancer (9, 39, 40). Wang et al. (41) showed that the serum albumin-to-globulin ratio (AGR) is an independent predictor of progression-free and cancer-specific survival. Patients with low AGR (<1.45) experienced poorer clinical outcomes. In another study, researchers evaluated inflammatory markers—CRP, haptoglobin, and albumin—in relation to PCa severity. However, only serum albumin was positively associated with overall mortality and higher-grade Gleason 4+3 tumors. Other inflammatory markers showed no association with survival or disease-specific mortality (26). Unlike BMI, GNRI includes serum albumin and therefore provides a more comprehensive assessment of nutritional risk (13).

Although advanced age is an established risk factor for poorer clinical outcomes in prostate cancer (42), these findings emphasize that nutritional and functional reserves significantly influence survival independent of chronological age. Previous studies have also reported an inverse association between GNRI and prostate cancer risk, further supporting the relevance of host nutritional status in disease progression (43). Additionally, lifestyle factors such as physical activity have been shown to influence prostate cancer development and clinical outcomes (44). This evidence underscores the necessity of a multidimensional approach to patient assessment.

With the increasing use of immunotherapies and novel androgen receptor signaling inhibitors (ARSIs) in prostate cancer treatment, the identification of biomarkers that predict treatment tolerance has become increasingly important. Low PNI or GNRI values, which indicate impaired nutritional and immune status, may correlate with a higher risk of treatment-related toxicity and reduced tolerance to these systemic therapies (45). For elderly patients with diverse comorbidities and varying degrees of treatment tolerance, incorporating nutritional status into clinical decision-making, alongside adverse pathological prognostic markers such as Gleason score and tumor volume, may facilitate the identification of individuals who could benefit from supportive interventions before the initiation of aggressive treatment (46).

This study has several limitations. Its retrospective, single-center design may introduce selection bias, and the absence of systemic inflammatory markers such as CRP restricts detailed evaluation of the interaction between inflammation and nutritional status. Furthermore, only baseline nutritional indices were assessed, whereas longitudinal measurements could more accurately capture the impact of dynamic changes in nutritional reserve on outcomes.

Methodologically, conventional ROC analyses were employed to assess the prognostic performance of PNI and GNRI. Time-dependent ROC or decision-curve analyses, as well as comparative ROC evaluations incorporating established tumor-related factors such as PSA level and Gleason score, were not conducted due to the retrospective design and limited number of events. Although PNI and GNRI remained independent predictors after multivariable adjustment, their incremental discriminative value beyond conventional clinical variables could not be fully quantified. Prospective, multicenter studies with extended follow-up and integrated molecular and nutritional models are necessary to address these limitations.

In clinical practice, the incorporation of PNI and GNRI into routine assessments may facilitate the identification of elderly patients with early-stage prostate cancer who are at increased risk of adverse outcomes and who could benefit from enhanced follow-up and structured nutritional support. While the retrospective design of this study precludes definitive conclusions regarding treatment modification based on these indices, the findings offer practical insight into physiological reserve and treatment tolerance among patients with diverse comorbidities.

Consistent with ESPEN recommendations, routine nutritional screening and early supportive interventions should be implemented for elderly patients undergoing prostate cancer treatment. The present findings support the use of PNI and GNRI as complementary prognostic tools that may enhance risk stratification and inform individualized management strategies, warranting further validation in prospective, multicenter studies.

## Data Availability

The raw data supporting the conclusions of this article will be made available by the authors, without undue reservation.
